# Screening for type 2 diabetes in a high-risk population: study design and feasibility of a population-based randomized controlled trial

**DOI:** 10.1186/1471-2458-12-671

**Published:** 2012-08-17

**Authors:** Bart Klijs, Suzie J Otto, Robert J Heine, Yolanda van der Graaf, Jan J Lous, Harry J de Koning

**Affiliations:** 1 Department of Public Health, Erasmus MC, P.O. Box 2040, 3000 CA, Rotterdam, Netherlands; 2 EMGO Institute for Health and Care Research, VU University Medical Center, Amsterdam, Netherlands and Eli-Lilly, Indianapolis, IN, USA; 3 Julius Center for Health Sciences and Primary Care, UMC Utrecht, Utrecht, Netherlands; 4 Star-Medical Diagnostic Center, Rotterdam, Netherlands

**Keywords:** Early detection, Screening, Type 2 diabetes, Abdominal obesity, Waist circumference

## Abstract

**Background:**

We describe the design and present the results of the first year of a population-based study of screening for type 2 diabetes in individuals at high risk of developing the disease. High risk is defined as having abdominal obesity.

**Methods:**

Between 2006 and 2007, 79,142 inhabitants of two Dutch municipalities aged 40–74 years were approached to participate in screening. Eligible participants had a self-reported waist circumference of ≥80 cm for women and ≥94 cm for men, and no known pre-existing diabetes. Of the respondents (n = 20,578; response rate 26%), 16,135 were abdominally obese. In total, 10,609 individuals gave written informed consent for participation and were randomized into either the screening (n = 5305) or the control arm (n = 5304). Participants in the screening arm were invited to have their fasting plasma glucose (FPG) measured and were referred to their general practitioner (GP) if it was ≥6.1 mmol/L. In addition, blood lipids were determined in the screening arm, because abdominal obesity is often associated with cardiovascular risk factors. Participants in both arms received written healthy lifestyle information. Between-group differences were analyzed with Chi-square tests and logistic regression (categorical variables) and unpaired t-tests (continuous variables).

**Results:**

The screening attendance rate was 84.1%. Attending screening was associated with age at randomization (OR = 1.03, 95% CI 1.02-1.04), being married (OR = 1.57, 95% CI 1.33-1.83) and not-smoking currently (OR = 0.52, 95% CI 0.44-0.62). Of the individuals screened, 5.6% had hyperglycemia, and a further 11.6% had an estimated absolute cardiovascular disease risk of 5% or higher, according to the Systematic Coronary Risk Evaluation risk model. These participants were referred to their GP.

**Conclusions:**

Self-reported home-assessed waist circumference could feasibly detect persons at high risk of hyperglycemia or cardiovascular disease. Continuation of the large-scale RCT is warranted to test the hypothesis that targeted population-based screening for type 2 diabetes leads to a significant reduction in cardiovascular morbidity and mortality.

**Trial registration:**

ISRCTN75983009

## Background

Type 2 diabetes is a major public health problem affecting more than 285 million people worldwide [1]. Diabetes is not only associated with an increased risk of developing microvascular complications [2], but also with a high risk of cardiovascular [3] and all-cause mortality [4]. Because of these complications, diabetes imposes a significant burden on the quality of life of the patient and on the health care system, and reduces the number of healthy life years. Diabetes prevention is, therefore, a major concern, and both diabetes and public health organizations worldwide have expressed the need for diabetes screening in asymptomatic individuals [5-10].

Type 2 diabetes meets many of the criteria for screening which were formulated by Wilson & Jungner [11] to aid the decision regarding whether or not to introduce a population-based screening program. Type 2 diabetes is an important health problem that can be diagnosed by means of simple, non-invasive and acceptable screening tests [12,13]. The onset of the disease is estimated to occur 9–12 years before clinical diagnosis [14]. Effective treatment options, either lifestyle modifications or medical treatment, are available for asymptomatic individuals [15]. Recently, the multicenter Anglo-Danish-Dutch Study of Intensive Treatment in People with Screen Detected Diabetes in Primary Care (ADDITION-Europe) demonstrated that early intensive management of screen-detected type 2 diabetes was associated with a non-significant relative reduction in the incidence of cardiovascular events after 5-years of follow-up compared with the screen-detected type 2 diabetes cases receiving usual care [13,16]. However, the effectiveness of screening as a means of prevention of diabetes has not yet been established [6,9,10].

Resulting from the epidemic growth of overweight, obesity and abdominal obesity, the number of newly diagnosed type 2 diabetes cases is expected to grow sharply in the coming years (439 million in 2030, [1]), which constitutes a further threat to public health [1,17]. Waist circumference has been put forward as first-step screening tool for the identification of undiagnosed diabetes, because there is strong evidence that this anthropometric measure gives better prediction of incident diabetes than body mass index (BMI) [18-21].

The current population-based randomized controlled trial (RCT) was set up to (a) assess the performance of waist circumference measurement as a first-step screening tool to identify individuals at high risk of developing diabetes; (b) examine the effectiveness of type 2 diabetes screening; and (3) ascertain whether early detection and treatment of type 2 diabetes results in a reduction and/or prevention of related morbidity and mortality compared with not offering screening. This paper describes the design and feasibility of this population-based RCT.

## Methods

### Study population

Participants were recruited among the inhabitants (males and females aged 40–74 years) of the working area of two Municipal Health Services in the Southwest Region of the Netherlands. Their name and address information was obtained from municipal authority records. An invitation letter together with the study material, comprising of an information brochure, a consent form, a tape measure and a questionnaire were sent by mail to the target population. The questionnaire contained questions on demographic features such as marital status and education, self-perceived health, weight and height, lifestyle, symptoms and diabetes related risk factors, family history of diabetes and personal history of selected diseases, including cardiovascular diseases, stroke, and diabetes.

The individuals were requested to measure their waist circumference twice with the tape measure sent to their home addresses and fill out their measurements on the questionnaire, in addition to data on sociodemographic characteristics, lifestyle and risk factors. After completion of the questionnaire, the potential participants returned it in the enclosed postage-free reply envelope. Eligibility, which was based on the absence of pre-existing diabetes and a self-reported waist circumference indicating abdominal obesity, was ascertained upon receipt of the questionnaires. Abdominal obesity was defined as a waist circumference of ≥80 cm for women and ≥94 cm for men, following the cut-off points of the International Diabetes Federation [8].

The validity of the self-reported waist circumference was assessed in a subset of 160 persons by comparing the self-measurements with measurements taken by a trained nurse. The Cohen's kappa, which was calculated as a proxy for the reliability between self-reported and professionally-measured waist circumference, was an acceptable 0.64.

**Figure 1:**
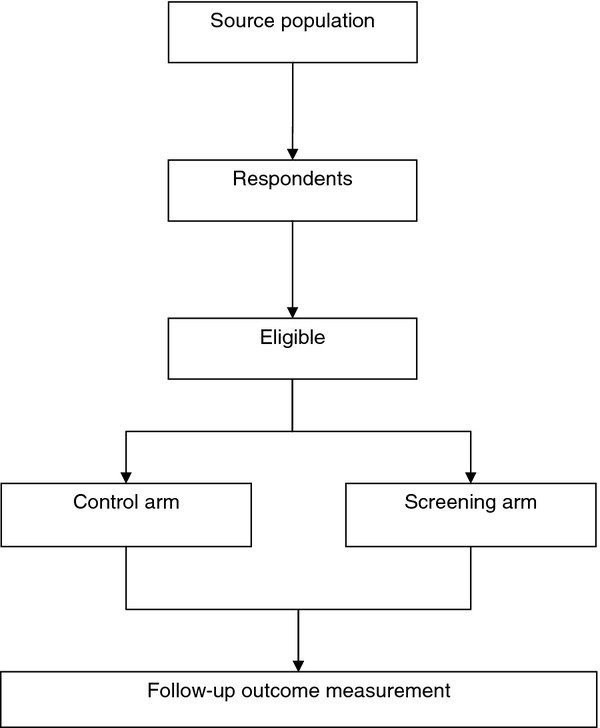
Flowchart of the screening trial.

A flowchart of the study is presented in Figure 1. The study protocol and materials were approved by the Medical Ethical Review Committee of Erasmus MC and the RCT was registered with Current Controlled Trials (ISRCTN75983009).

### Randomization

Eligible consenting respondents were stratified by gender and individually randomized at a 1:1 ratio to the intervention (screening) arm or the control arm using a random number generator. Participants in the screening arm received an invitation to attend screening, which consisted of fasting plasma glucose (FPG) measurement and information about the importance of a healthy lifestyle (Netherlands Nutrition Centre). Those in the control arm only received the healthy lifestyle information.

### Intervention

FPG was used as the screening criterion in accordance with the Dutch College of General Practitioners’ guidelines [22]. The FPG cut-off values were 7.0 mmol/L or higher for diabetes and between 6.1 and 6.9 mmol/L for Impaired Fasting Glucose (IFG) [22]. As dyslipidemia are often present in overweight and obese subjects, and it is clinical practice to measure blood lipids in patients with diabetes, we opted to additionally measure serum lipids (total cholesterol, high-density lipoprotein, HDL, cholesterol and triglycerides). FPG concentrations were determined using the hexokinase method. Fasting serum total cholesterol, HDL cholesterol and triglycerides were quantified using an enzymatic colorimetric method. All analyses were performed on the Beckman AU2700 chemical analyzer (Beckman Coulter Nederland BV, Woerden, Netherlands), using kits supplied by Beckman. Low-density lipoprotein (LDL) was calculated using the Friedewald formula [23].

Individuals with a FPG level of 7.0 mmol/L or higher were referred to their general practitioners (GP) for confirmatory diagnostic testing and treatment, which included glycemic control by lifestyle intervention or antihyperglycemic medication (oral agents and eventually insulin if necessary), and management of any present cardiovascular risk factors. Individuals with IFG were also referred to their GP for FPG monitoring and treatment of possible cardiovascular risk factors according to the GP guidelines. Participants were told their blood glucose level; this information was also given to their GP. Normoglycemic individuals with an estimated absolute cardiovascular disease risk of 5% or higher, according to the Systematic Coronary Risk Evaluation (SCORE) risk model, were also referred to their GP [24]. The SCORE risk estimates are calculated based on age, blood pressure level, current smoking status and the ratio of total cholesterol to HDL cholesterol.

Because all participants in this RCT received the same disease information, those assigned to the control arm might take opportunistic testing after learning about the simplicity of the screening test. Therefore, FPG testing in the control arm was monitored through their GPs and linkages with laboratory databases, similar to a previous prostate cancer screening trial [25].

### Outcome measures

The primary endpoint of the trial was the first occurrence of a fatal or non-fatal cardiovascular event within the follow-up period after randomization. A cardiovascular event was defined as non-fatal coronary heart disease, non-fatal stroke or death from any disease of the circulatory system (among diabetic cases and those at intermediate to high cardiovascular risk). Estimates of prevented microvascular complications are also important, but this study primarily focuses on macrovascular complications, because these are by far the most important contributor to premature death among patients with diabetes. A secondary analysis will include the primary non-fatal endpoint, as well as non-cardiovascular diabetes-related morbidity and mortality, all-cause mortality and cardiovascular interventions (e.g. revascularization).

Intermediate endpoints include the prevalence/incidence of IFG and newly screen-detected type 2 diabetes, screening performance (attendance, referral and detection rates, and test characteristics), number of individuals with an absolute cardiovascular disease risk of 5% or higher (intermediate to high), and temporal changes in the levels of blood parameters (glucose, HbA_1_c, lipids and blood pressure).

### Follow-up and data collection

For each participant, a minimum follow-up period of 10 years is planned. During follow-up, information on the time of diabetes diagnosis, cardiovascular risk profiles, cardiovascular morbidity, diabetic complications and use of medication will be obtained from GPs and from the National Hospital Discharge Register. Data on cardiovascular risk profiles, cardiovascular morbidity and medication use will also be collected for those referred to their GP based on their absolute cardiovascular disease risk. Vital status will be ascertained through record linkage with the Cause of Death Registry of Statistics Netherlands.

### Sample size

In the sample size calculations we estimated the minimum number of participants required to identify a statistically significant 20% reduction in non-fatal and fatal cardiovascular events in the screening compared with the control arm. The expected rate of the composite outcome of cardiovascular events in an aging cohort of 40–74 years at entry was estimated at 2% per year, based on 2003 age and gender specific data obtained from the Hospital Admission Registry for non-fatal events and Statistics Netherlands for fatal events. Using age-specific abdominal obesity prevalence data, the number of events was estimated in the sub-population with abdominal obesity [26]. Using the method developed by Baan and coworkers [27] to assess the diabetes-related mortality in the Dutch population, we additionally accounted for the prevalence of pre-existing diabetes among individuals with abdominal obesity [6], the relative risk of developing type 2 diabetes in this group [26], and sex-specific hazard rates for cardiovascular events. We estimated that the required number of participants per study arm over 10 years was 30,885 to show a reduction of 20%, with a power of 80% and a two-sided significance level of 5%, assuming a contamination rate of 10% in the control arm, as previously reported for prostate-specific antigen screening [25].

### Feasibility phase

In the first year of the trial, we examined the feasibility of home-assessed and self-reported waist circumference measurements as a first-step screening tool for recruiting individuals at high risk. The sociodemographic characteristics of the respondents and the intermediate endpoints, particularly attendance and detection rate were assessed. Socioeconomic status was based on 2006 social status ranking of the Social and Cultural Planning Office. The ranking, from 1 (high status) to 3965 (low status) is estimated based on income, employment status and level of education in the households within postal code areas.

In 2006 and 2007, 79,142 inhabitants of the Dutch municipalities of Capelle aan den IJssel and Dordrecht, in the age group 40–74 years were invited to participate. The mailings were sent out in two different ways. Half of the population received the complete study material, consisting of a letter of invitation, information brochure, tape measure and consent form (one-step screening approach). The other half first received a letter of invitation containing a brief introduction to the study, an explanation of how to measure their waist size, and a tape measure. Individuals who responded and who were judged potentially eligible were then sent an accompanying letter, information brochure and the consent form (two-step screening approach). We aimed to find out which method produced the greatest response from people at high risk, and what the costs of each strategy were. In either approach strategy, those eligible for participation were randomized only if they gave their informed consent.

### Statistical analysis

Differences between respondents and non-respondents and between the control arm and the screening arm were compared using Chi-square tests for categorical variables and unpaired *t*-tests for continuous variables.

To examine the effect of the variables age, waist size (continuous) and gender, marital status, education, smoking status, family history and country of birth (categorical) on the odds of attending screening, univariate logistic regression was used and then a multivariate model was constructed with the variables that had a univariate *p*-value of ≤0.10. The results are presented as odds ratio (OR) and corresponding 95% confidence interval (CI).

All analyses were performed using SPSS version 17 (SPSS, Chicago, IL, USA). A *p*-value of <0.05 was considered statistically significant.

## Results

A total of 20,578 of the 79,142 individuals approached in the municipalities of Capelle aan den IJssel and Dordrecht returned the questionnaire (26% response rate). The sociodemographic characteristics of the respondents and non-respondents are presented in Table 1. Compared with the non-responders, the responders in both municipalities were older, predominantly females and born in the Netherlands. In both municipalities there was a tendency of higher response rates among those of middle to high social status. The overall response among people born in the Netherlands was 27.8%, and among immigrants was 15.3%. Of the major ethnic groups, the lowest response was among people born in Morocco (7.8%), compared with those born in Turkey (12.7%), the Dutch Antilles (16.4%) and Surinam (19.8%).

**Table 1:** Comparison between respondent and non-respondent to initial questionnaire in the two municipalities

	**Capelle aan den IJssel**				**Dordrecht**			
	**Total**	**Responders**	**Non-responders**	P-value*	**Total**	**Responders**	**Non-responders**	P-value*
	**N = 29,163**	**n = 7,779**	**n = 21,385**		**N = 49,979**	**N = 12,799**	**N = 37,180**	
**Age at mailing,***median, yrs†*	53.6	55.1	52.9	0.000	54.1	55.9	53.5	0.000
**Gender, male,***%*	47.9	45.9	48.6	0.000	49.6	46.5	50.7	0.000
**Country of birth,***%*								
Netherlands	82.9	88.3	80.9	0.000	84.0	89.9	81.9	0.000
Other	17.1	11.7	19.1		16.0	10.1	18.1	
* Western country*	*6.6*	*4.7*	*7.2*		*6.3*	*4.8*	*6.8*	
* Non-Western country*	*10.6*	*7.0*	*11.8*		*9.7*	*5.3*	*11.2*	
**Social Status,***%*								
High	30.3	39.2	27.0	0.000	0.8	0.6	0.9	0.000
Middle high	5.1	3.8	5.6		43.1	51.3	40.3	
Middle low	42.8	33.2	46.4		23.6	21.7	24.3	
Low	21.8	23.8	21.0		32.5	26.4	34.6	

*Chi-square test for comparison of proportions and unpaired t-test for comparison of continuous variables between responders and non-responders.

†Age at mailing of study material.

**Figure 2:**
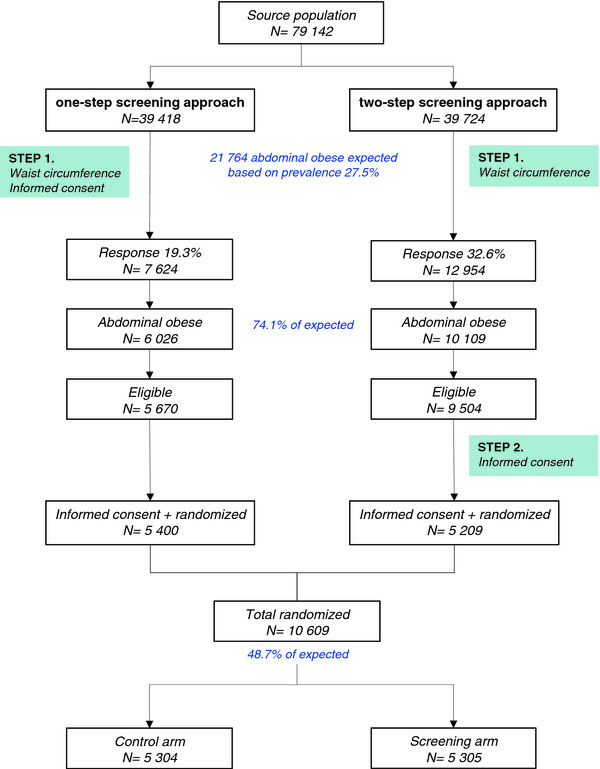
Outcome screening procedure with the one-step and two-step screening methods.

Assuming a prevalence of 27.5% [26], 21,764 out of 79,142 individuals approached for participation were expected to have abdominal obesity. In total, 10,609 individuals, which is 48.7% of the expected abdominally obese population, were eligible and randomized as part of the trial (Figure 2). The one-step screening strategy, i.e. asking consent for participation before ascertainment of eligibility, yielded a slightly higher proportion of participants than the two-step strategy. Consequently, the costs of recruitment per participant were lower for the one-step approach than for the two-step approach: the costs were €5.90 and €4.60 (USD 7.55 and 5.89) per participant in the screening and the control arm in the one-step procedure, and €6.67 and €5.43 (USD 8.58 and 6.98), in the two-step procedure.

The characteristics of the participants randomized in the screening (n = 5305) and in the control arm (n = 5304) are presented in Table 2. Among those in the screening arm invited for FPG measurement, 4457 participants actually attended screening (84.1% attendance rate). Screening attendance was significantly related to age at randomization (OR = 1.03, 95% CI 1.02-1.04, p < 0.001), being married (OR = 1.69, 95% CI 1.44-1.97, p < 0.001), not-smoking currently (OR = 0.47, 95% CI 0.40-0.56, p < 0.001) and born in the Netherlands (OR = 1.34, 95% CI 1.05-1.70, p = 0.017). In the multivariate analysis, only country of birth was no longer statistically significant.

**Table 2:** Characteristic of the control arm and the screening arm, and univariate and multivariate odds ratios and 95% confidence intervals for determinants of attending screening

	**Control arm n = 5304**	**Screening arm n = 5305**	P-value*	**Screening attendees**	**Screening non-attendees**	**Univariate OR (95% CI)†**	**Multivariate OR (95% CI)†**
**Age at randomization**, *mean (range), yrs*	56.7 (39.9 – 75.9)	56.6 (39.9 – 76.0)	0.299	57.0	54.4	1.03 (1.02-1.04), p < 0.001	1.03 (1.02 – 1.04), p < 0.001
**Gender**, *male, %*	43.9	43.7	0.778	43.4	44.9	0.94 (0.81-1.09), p = 0.422	
**Marital status**, *married, %*	74.4	73.6	0.379	75.4	64.5	1.69 (1.44-1.97), p < 0.001	1.57 (1.33 – 1.83), p < 0.001
**Highest education completed,***%*							
Primary	9.5	9.6	0.917	9.2	11.2	0.89 (0.68-1.16), p = 0.400	
Lower secondary	40.8	39.9		40.3	38.2	1.14 (0.95-1.37), p = 0.169	
Upper secondary	24.2	24.8		25.3	23.1	1.18 (0.96-1.45), p = 0.126	
Tertiary	24.4	24.6		24.3	26.3	*Reference*	
**Smoking status,***current smoker, %*	18.7	19.8	0.389	17.6	31.1	0.47 (0.40-0.56), p < 0.001	0.52 (0.44 – 0.62), p < 0.001
**Waist circumference,***mean, cm*	96.2	96.6	0.214	96.6	96.6	1.00 (0.99-1.01), p = 0.377	
* Females 80–87, %*	*25.3*	*25.3*	*0.266*	*24.6*	*28.7*		
* > = 88, %*	*74.7*	*74.7*		*75.4*	*71.3*		
* Males 94–101, %*	*46.4*	*46.5*	*0.282*	*47.2*	*43.0*		
* > = 102, %*	*53.5*	*53.5*		*52.6*	*57.0*		
**Diabetes family history**, *%*	27.2	26.4	0.350	26.5	25.8	1.04 (0.88-1.23), p = 0.674	
**Country of birth,***%*							
Netherlands	91.4	91.1	0.583	91.5	88.9	1.34 (1.05-1.70), p = 0.017	1.20 (0.94-1.54), p = 0.138
Other	8.6	8.9		8.5	11.1		
* Western country‡*	*4.0*	*4.0*		*4.1*	*3.5*		
* Non-western country*	*4.6*	*4.9*		*4.4*	*7.5*		
**Screening outcome**							
Attendance rate, n, (%)	–	4457 (84.0)					
Impaired fasting glucose, n (%)	–	170 (3.8)					
Diabetes, n (%)	–	81 (1.8)					
SCORE† > =5%,	–	518 (11.6)					

* Chi-square test for comparison of proportions and unpaired t-test for comparison of continuous variables between responders and non-responders.

† OR, odds ratio; CI, confidence interval; SCORE, Systematic COronary Risk Evaluation.

‡ European countries (excluding Turkey), North America, Oceania, Indonesia or Japan.

**Figure 3:**
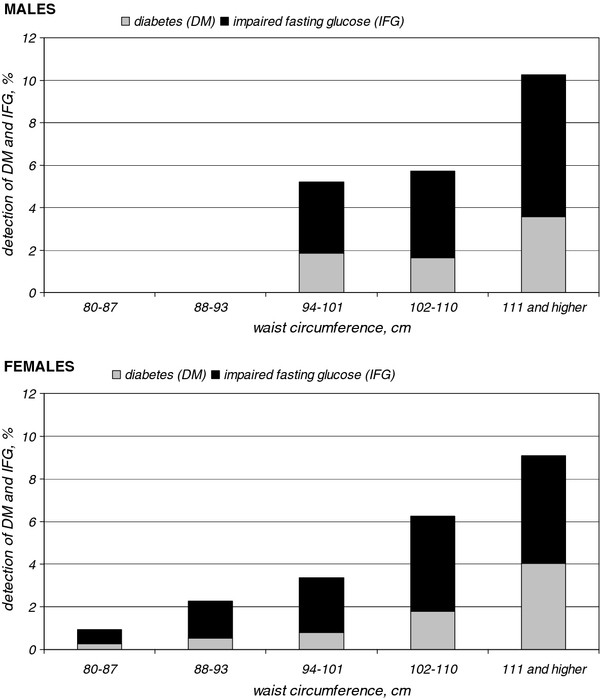
The distributions of the impaired fasting glucose and diabetes rates across males and females according to their waist circumference.

In total, 251 persons were found to have glucose levels ≥6.1 mmol/L at screening (5.6%), which prompted a referral to their GP. The distributions of the IFG and diabetes detection rates across males and females according to their waist circumference are presented in Figure 3. Based on their lipids, 518 screened participants (11.6%) had a moderate to high cardiovascular risk (SCORE risk ≥5%) and were referred to their GP.

## Discussion

The availability of effective treatment options and the fact that screening can advance the moment of diagnosis logically lead to the suggestion that systematic screening for type 2 diabetes will reduce cardiovascular-related morbidity and mortality. However, an RCT is required to confirm this hypothesis and assess whether screening is cost-effective. The aim of our extensive feasibility assessment was to examine the performance of home-assessed and self-reported waist circumference measurements as first-step screening tools for recruiting high-risk individuals. Self-reported waist circumference proved to be a feasible method for detecting persons at risk of IFG, type 2 diabetes and/or high cardiovascular risk. Given the percentage of newly diagnosed diabetes by waist circumference, a cut-off value of ≥94 cm could be used for males, and of ≥88 cm could be used for females. Because the one-step strategy, whereby consent for participation was asked before proven eligibility, yielded slightly more high-risk persons than the two-step strategy, while saving the costs of an extra mailing, the one-step strategy is the preferred recruitment method.

The feasibility of using abdominal obesity to detect persons with unknown diabetes or at high cardiovascular risk has previously been assessed in different settings [28-30]. Van den Donk et al. [29] reported a 27.5% detection rate for metabolic syndrome in 1721 individuals who were abdominally obese. In a validation study, Korhonen et al. [30] showed that 95% of all new cases of diabetes and 84% of all new cases of IFG could be identified based on the presence of abdominal obesity. Although specificity could not be assessed in our study, in line with the aforementioned studies, we found self-reported home-assessed abdominal obesity to be a feasible tool for detecting persons with unknown diabetes and/or who were at high cardiovascular risk.

In the Netherlands, diabetes screening has previously been examined in at least two studies. In the ADDITION Netherlands study (2002–2004), population-based screening was performed via GPs [31]. Of all 56,978 persons who were invited to participate based on the Symptom Risk Questionnaire (SRQ), 17,883 (30%) were screened. The SRQ contains questions on age, sex, BMI, family history of diabetes, frequent thirst, use of antihypertensive medication, shortness of breath, claudication, and cycling [32]. The prevalence of newly detected diabetes and IFG/impaired glucose tolerance was 3.3% and 5.7%, respectively. In the Dutch Hoorn screening study, 11,679 individuals were invited to participate, of whom 7736 completed the SRQ [33]. Of the 7736, 2885 (37.3%) had a high-risk profile and underwent a capillary glucose measurement and 217 new cases of diabetes were detected. Our study differs from those two studies with respect to the study population (e.g. age, definition of “high risk”) and the method used to verify diabetes, which may explain the lower percentages of newly detected IFG and diabetes in our study. In addition, the lower percentages may be related to improved efforts to detect and treat unknown diabetes [34].

The response rate following the initial invitation to provide waist circumference measurements was only 26%, which could raise doubts about the success in reaching the target group and the feasibility of the study. However, this 26% is a proportion of all of the individuals contacted, not the actual study target group (people with abdominal obesity who are at high risk). The eventual proportion of anticipated high-risk individuals who agreed to undergo randomization was 49%. Other projects involving the mailing of questionnaires also show that the response rate can vary markedly depending on the individual and the age group contacted. This makes it difficult to predict response rates. The response rate in a cluster-RCT of screening for delays in language development conducted among 10,000 parents of three-year-old children was 75% [35]. When invitations to a trial of screening for prostate cancer were sent to 40,000 men, the response rate was 40% [36]. Sending out general questionnaires through local health authorities for a lung cancer screening project typically produced response rates of around 32% [37]. Our observation of 49% of the target high-risk population being randomized falls within these previously reported rates.

### Limitations

As aforementioned, only 49% of the expected obese population consented to participate. In the course of the trial, efforts should be undertaken to further improve participation rates, for instance, by sending reminders. The study materials were written in easily understandable Dutch (targeted at level B1, advanced low, of the Common European Framework of Reference for Languages [38]), to ensure that the study information was accessible to people with a low level of educational attainment and to reach the immigrant population. Advertisements were placed in local newspapers in the two study towns around the time that the letters were being sent out. However, the participation rates among individuals with a low social status or from non-Dutch origin were low and could probably also be improved by adapting the language to a lower level or by translation to meet the specific requirements of these groups. The involvement of people from local immigrant populations in the recruitment process may prove to be effective.

In our study, we chose to use waist circumference as the first-step screening tool rather than a questionnaire. We did collect information using the nine questions of the Dutch-validated SRQ, which was used as first screening step in the Hoorn Study [32] and the ADDITION Netherlands study [31], but we did not use this information. Several other risk questionnaires have been developed to identify individuals with increased risk of developing type 2 diabetes, of which the FINDRISC tool was found to be the best available for use in clinical practice [39]. However, this tool has not yet been validated in a Dutch population. Chamnan and colleagues recently calculated that using anthropometric measures (BMI ≥25 kg/m2 or waist circumference >94 cm in men and >80 cm in women) showed slightly higher sensitivity and discriminatory ability compared with inviting individuals based on the FINDRISC cut off or the Cambridge risk score [40].

## Conclusions

Self-reported home-assessed waist circumference proved to be a feasible method for detecting persons at high risk of hyperglycemia, but further work is necessary to increase the uptake of this anthropometric measure as a first-step screening tool. Continuation of this large-scale RCT is warranted to establish whether diabetes screening leads to a significant reduction in cardiovascular morbidity and mortality. The results will contribute to the evidence for or against the provision of screening for type 2 diabetes, and to the development of a strategy for the identification and selection of the population at risk.

## Competing interests

The authors declare that they have no competing interests.

## Authors' contributions

HK contributed to the conception and design of the study, guided the data collection, contributed to interpretation of data, and was involved in critically revising the manuscript. RH and YG contributed to the study design and interpretation of data. SO and BK contributed to the design of the study, data collection and analysis, interpretation of data, and were involved in drafting the manuscript. All authors read, critically revised the drafts and approved the final version of the manuscript for publication.

## Pre-publication history

The pre-publication history for this paper can be accessed here:

http://www.biomedcentral.com/1471-2458/12/671/prepub
